# MRI Evaluation of Soft Tissue Tumors and Tumor-Like Lesions of Extremities

**DOI:** 10.7759/cureus.37047

**Published:** 2023-04-02

**Authors:** Shreeya Goyal, Varsha Rangankar, Sanika Deshmukh, Aparna Prabhu, Johnson S

**Affiliations:** 1 Radiodiagnosis, Dr. D. Y. Patil Medical College, Hospital & Research Centre, Pune, IND; 2 Community Medicine, Dr. D. Y. Patil Medical College, Hospital & Research Centre, Pune, IND

**Keywords:** tumor mimics, malignant, benign, sarcoma, soft tissue tumors, extremity

## Abstract

Aim: The current study aimed to evaluate the use of magnetic resonance imaging (MRI) in the diagnosis of extremity soft tissue tumors and tumor-like abnormalities.

Methods: This prospective observational study of 71 patients with soft tissue lesions of extremities was conducted at a tertiary hospital and teaching center in western India after obtaining Institutional Ethical Committee (IEC) clearance. All patients underwent an MRI of the region of interest on Siemens Magnetom Vida 3 Tesla MRI (Erlangen, Germany). MRI findings and diagnosis were correlated clinically and with histopathological examination.

Results: A total of 71 patients (49 males and 22 females) in the age group of six to 90 years were included in our study. Out of 44 patients with soft tissue tumors, the most common lesion was neurofibroma (18.1%), followed by lipoma and undifferentiated sarcoma (9.1% each). Liposarcoma, myxoid liposarcoma, giant cell tumor of the tendon, pigmented villonodular synovitis, and schwannoma were seen in 4.5% of patients each. The soft tissue tumor-like lesions were seen in 27 (38%) patients, the most common being slow-flow vascular malformation, which was seen in 9/27 (33%) patients. The second most common pathology was actinomycosis, seen in four (14.8%) patients. Out of 44 patients with soft tissue tumors, 27 (61.4%) were benign and 17 (38.6%) were malignant. Tumor size of more than 5 cm was more commonly seen in malignant tumors (70.5%) than benign tumors (40.7%). The smooth margin was more common in benign tumors (70.3), while most malignant tumors (70.5%) had irregular or lobulated margins. Heterogenous enhancement was more common in malignant tumors (82.3%) than benign tumors (62.9). The odds of a benign histopathological diagnosis for a tumor suspected to be benign by MRI were 93.75 times higher than the odds of a benign histopathological diagnosis for a tumor suspected to be malignant by MRI.

Conclusion: MRI is extremely useful in the evaluation of different soft tissue masses and helps in evaluating the characteristics of the masses, their extent and relationship to surrounding structures, and bone destruction, multiplicity, composition, and enhancement pattern. The systematic imaging analysis approach helps to differentiate a benign lesion from a malignant lesion and also in differentiating various soft tissue tumor mimics.

## Introduction

Soft tissue originates from the mesenchyme, which differentiates into fat, skeletal muscles, blood vessels, fibrous tissue, and peripheral nerves during development [1]. Soft tissue lesions comprise a wide range of lesions from non-neoplastic to benign to malignant tumors. Soft tissue tumors are a diverse collection of mesenchymal neoplasms that exhibit a wide spectrum of differentiation. Although soft tissue components that make up the lesion are used to define soft-tissue malignancies histologically, this does not always suggest that the tumor originates from that tissue [1]. The widely used World Health Organization (WHO) classification system for soft tissue tumors offers consistency in the reporting and treatment of different tumors and reactive processes [2].

The most common overall soft tissue lesion is a lipoma and it is very important to differentiate a benign lipoma from well-differentiated liposarcoma on imaging. The presence of fat may be minimal and not visible in dedifferentiated, myxoid, and pleomorphic types of liposarcoma. Benign peripheral nerve sheath tumors (PNSTs) include two common types of tumors: schwannoma and neurofibroma. Malignant PNSTs are less common and are usually associated with type I neurofibromatosis [1]. Soft tissue tumors of the extremities most frequently occur in the thigh and buttocks, where they are also more likely to be malignant. A tumor deeper than 5 cm located proximally in the limb of a patient older than 50 years has a 50% chance of being malignant [3]. The benign vascular lesion known as a hemangioma is frequently found in the first decade of life and histologically mimics normal blood arteries. Proliferative lesions also known as hemangiomas typically develop after birth as a result of pathologic angiogenesis [4,5]. These tumors experience a proliferative period in the first few years of life during which they develop quickly before involuting over the following years.

Patients with suspected soft tissue masses are mainly referred to radiology for localization, characterization, and assessment of the extent of the lesion to decide the treatment plan. Soft tissue tumors also need to be differentiated from tumor-mimicking lesions like ganglia, hematomas, foreign body granulomas, and vascular malformations. Ultrasound (US) is the initial investigation in the evaluation of soft tissue masses and can provide information on the size and anatomical location of the lesions, differentiate solid and cystic lesions, and also reveal vascularity with the use of the color Doppler [5]. However, imaging modalities like X-rays, US, and, computed tomography have limitations and can be inconclusive in many cases [1]. Magnetic resonance imaging (MRI) with its multiplanar imaging, excellent spatial resolution, soft tissue characterization, and lack of radiation is the preferred imaging modality in the evaluation of soft tissue lesions, offering clinically important information and helping to decide the treatment protocol [6-9]. MRI also helps in the surgical approach by demarcating affected from unaffected regions, tumors from adjoining fat and muscles, and the lesions from adjacent neurovascular bundles. MRI helps in the timely diagnosis of soft tissue tumors as delay in diagnosis and hence treatment can worsen the prognosis of malignant soft tissue tumors because of local complications, greater morbidity after surgical treatment, and a higher rate of metastasis [10].

The present study assesses the role of MRI in the evaluation of soft tissue tumors and tumor-like lesions of extremities.

## Materials and methods

This prospective observational study was conducted at Dr. D. Y. Patil Medical College, Hospital & Research Centre, Pimpri, Pune from September 2020 to August 2022. Seventy-one patients with soft tissue lesions of their extremities underwent MRI on Siemens Magnetom Vida 3 Tesla MRI scanner (Erlangen, Germany). Institutional Ethical Committee (IEC) clearance was obtained before the start of the study from the Institutional Ethics Sub-committee, Dr. D. Y. Patil Medical College, Hospital & Research Centre, Pimpri, Pune (Research Protocol No.: IESC/PGS/2020/169). Informed and written consent was obtained from all the patients or guardians of minor patients. Inclusion and exclusion criteria are mentioned in Table 1 and Table 2, respectively.

**Table 1:** Inclusion criteria

Inclusion criteria
Patients of all age groups with clinically suspected soft tissue lesions in extremities.
Patients who are referred for MRI with a diagnosis of soft tissue lesion on any other imaging modality.

**Table 2:** Exclusion criteria

Exclusion criteria
Patients with a diagnosis of primary bone tumors with soft tissue involvement and lymph nodal masses.
Patients previously operated on for soft tissue lesions referred for follow-up or suspected recurrences.
Patients having cochlear implants, metallic foreign bodies, and non-MRI compatible orthopedic implants.
Patients with a history of claustrophobia.

MRI scan technique

Patient Positioning

The position of the patients was supine with head first or feet first directed toward the magnet as per the area of interest. The patients were asked not to move during the study and proper immobilization of the region of interest was performed.

Scan Planes Used

Axial, coronal, and sagittal planes were used. The orthogonal planes were chosen according to the location of the lesion. MRI sequences used in the study are mentioned in Table 3. All the lesions were evaluated with T1-weighted, T2-weighted, short tau inversion recovery (STIR), diffusion-weighted imaging (DWI), gradient recalled echo (GRE), and non-contrast and post-contrast-enhanced fat-suppressed T1-weighted images. MRI sequences scan planes were selected and modified based on the region of interest.

**Table 3:** MRI sequences used SE: spin echo; STIR: short tau inversion recovery; FS: fat-suppressed; T2*: T2 star (effective T2).

MRI sequences used (selected and modified as per the region of interest)
Axial T1-weighted
Axial T2-weighted fast SE
Axial STIR
Coronal, sagittal, or oblique longitudinal T1-weighted
Coronal, sagittal, or oblique longitudinal STIR
Diffusion-weighted imaging (DWI)
Gradient recalled echo (GRE) T2*-weighted
Axial non-enhanced fat-suppressed (FS) T1-weighted SE
Axial contrast-enhanced FS T1-weighted SE
Coronal, sagittal, or oblique longitudinal contrast-enhanced FS T1-weighted SE
Dynamic contrast-enhanced magnetic resonance angiography and three-dimensional FS T1-weighted sequence

MRI analysis

MRI images of all patients were analyzed and the following imaging features of the lesions were noted.

Location

Appendicular and trans-spatial locations were analyzed. Lesions were localized to the specific site in extremities (upper limb, lower limb, wrist, knee, etc.) and also according to the level of involvement as originating from the skin, subcutaneous fat, muscles, and intramuscular plane.

Size

Size in maximum dimensions was categorized as >5 cm and <5 cm, which was kept as the reference of the large and small lesions, respectively.

Margin

The lesions were categorized into lesions with smooth margins, irregular margins, and lobulated margins.

Signal Characteristics

The signal intensity of the lesions was analyzed on T1-weighted images and T2-weighted images and fat-suppressed images for tissue characterization of the lesion. The main composition of the lesion was studied based on the signal characteristics on MRI sequences, for example, lesions with the predominant adipocytic component and fibrous component.

Contrast Enhancement Pattern

Two patterns of contrast enhancement were considered for the lesions: homogeneous and heterogeneous enhancement.

Specific Signs

Specific signs such as a split fat sign, fascicular sign, target sign, and dot-in-circle sign were recorded.

The tissue characterization of the lesion on MRI was done and also various imaging features like signal characteristics, presence of calcification, necrosis, hemorrhage, fat, etc., and presence of diffusion restriction were documented. Enhancement features and adjoining relations and extensions of the lesions were studied. All the MRI features were correlated and used to identify soft tissue tumors versus tumor-like lesions and to differentiate between benign and malignant pathologies. Correlations with histopathology, clinical findings, and other imaging modalities like X-ray, ultrasound, and computed tomography if available were done whenever required to reach a diagnosis. The data obtained were collected, compiled, and tabulated. All statistical calculations were done using statistical tools with Epi Info version 7.2.4 (CDC, Atlanta, Georgia).

## Results

A total of 71 patients with soft tissue masses in the age group of 6-90 years were included in our study (Table 4). Most of the patients were in the age group of 20-40 years comprising 30 (42%) cases, followed by 11 patients in the 60-70 years age group. The youngest case was a six-year-old male who underwent imaging for soft tissue mass in the elbow region. The study population had 49 males and 22 females with a male-to-female ratio of 2.2:1. Among the 71 patients, soft tissue mass swelling was the most common presenting feature seen in 57 (76%) patients, followed by pain seen in 42 (56%) patients (Table 5). Some common symptoms were localized redness, fever, and tingling sensation.

**Table 4:** Age and gender distribution

Age group	Male	Female	Total
Less than 10	3	0	3
10-19	5	2	7
20-29	11	4	15
30-39	9	6	15
40-49	5	4	9
50-59	3	2	5
60-69	8	3	11
70-79	4	1	5
80-89	1	0	1
Total	49	22	75

**Table 5:** Clinical presentation

Symptoms	Number (Total = 71)	Percentage
Swelling	57	76
Pain	42	56
Redness	8	10
Fever	5	6
Tingling	6	8

In the study population of 71, the most commonly affected region in the lower extremity was the thigh, seen in 19 (27%) cases, followed by the leg, seen in 12 (17%) cases. The most common location in the upper extremity was the arm, seen in seven (10%) cases (Table 6).

**Table 6:** Appendicular localization of the lesions

Appendicular location	Number, n = 71 (%)	Benign	Malignant
Upper limb			
Shoulder	1 (1)	1	0
Arm	7 (10)	5	2
Elbow	3 (4)	2	1
Forearm	4 (6)	4	0
Wrist	2 (3)	3	0
Hand	6 (8)	6	0
Total	23 (32.4)		
Lower limb			
Thigh	19 (27)	10	9
Knee	8 (11)	7	1
Leg	12 (17)	5	7
Ankle	3 (4)	2	1
Foot	6 (8)	5	1
Total	58 (67.6)		

**Figure 1:**
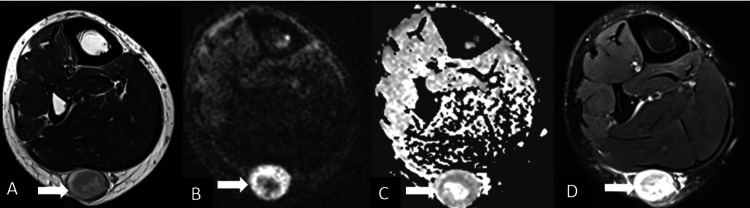
MRI of the calf showing neurofibroma MRI of the calf region in a 30-year-old male with progressively increasing swelling of the right calf for four months showing a well-defined lesion in the subcutaneous plane in the posterior aspect of the leg. The lesion is heterogeneously hyperintense on T2-weighted images with a thin peripheral hypointense rim (A) with no significant diffusion restriction (B, C) and moderate to strong heterogeneous contrast enhancement (D). Diagnosis: neurofibroma.

**Figure 2:**
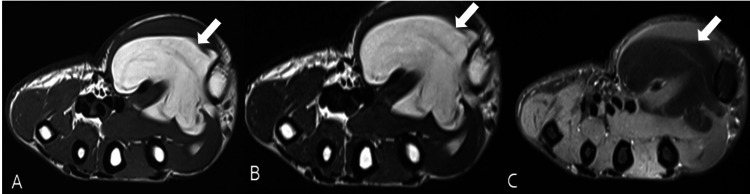
MRI of the wrist showing lipoma Axial MRI images of the wrist in a 35-year-old female with swelling in the right thenar region showing a well-defined lobulated lesion localized in the intermuscular plane deep to the thenar muscles, which appears hyperintense on T1-weighted (A) and T2-weighted (B) images and showing signal suppression on two-dimensional (2D) fast spin-echo (FSE) fat-suppressed proton density image (C). Diagnosis: lipoma.

**Figure 3:**
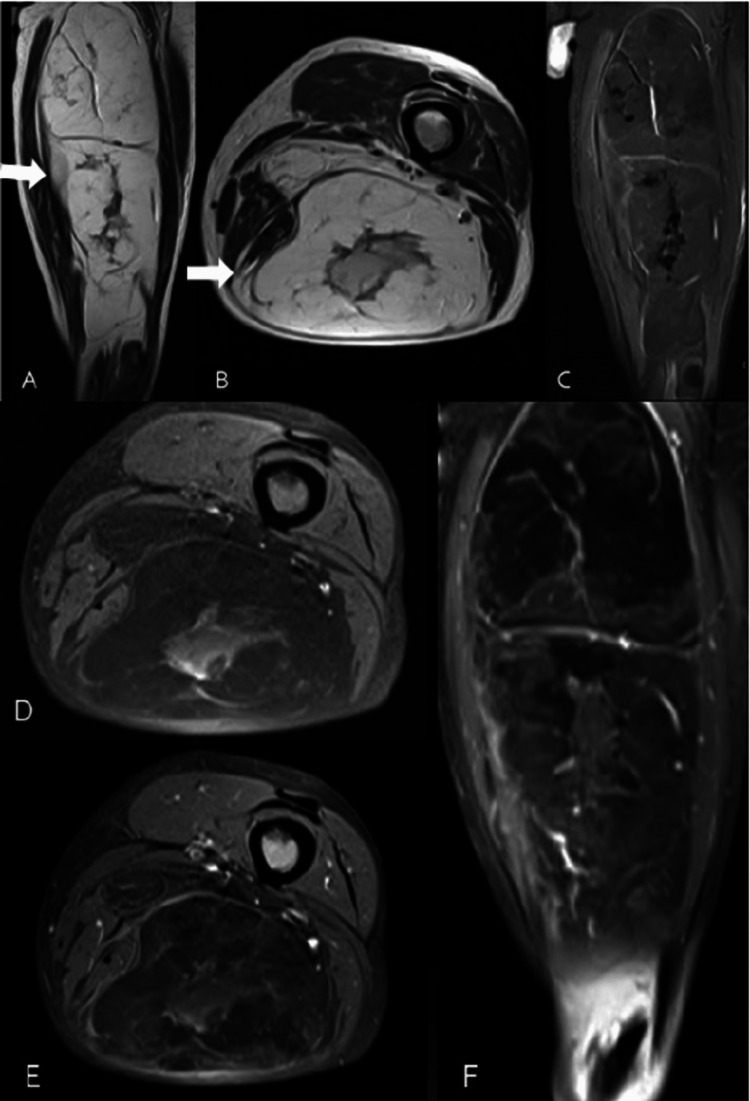
MRI of the thigh showing liposarcoma MRI of the left thigh in a 66-year-old male with gradually increasing thigh swelling that shows a well-defined capsulated large soft tissue lesion localized in the intramuscular plane of the posterior aspect of the thigh appearing hyperintense on T1-weighted (A) and T2-weighted images (B) with signal suppression on short tau inversion recovery (STIR) (C) images. The lesion shows heterogeneous post-contrast enhancement with the enhancement of the septae and soft tissue component on T1 fat-suppressed (T1FS) images (D-F). Diagnosis: liposarcoma.

Soft tissue tumors were seen in 44 (62%) out of 71 patients included in our study (Table 7). Out of 44 patients with soft tissue tumors, the most common lesion was neurofibroma, seen in eight (18.1%) cases (Figure 1). The second common type of tumor was a lipoma (Figure 2) and undifferentiable sarcoma, which were seen in four (9.1%) patients each. Liposarcoma (Figure 3), myxoid liposarcoma, giant cell tumor (GCT) of the tendon, pigmented villonodular synovitis (PVNS), and schwannoma were each seen in two (4-5%) patients.

**Table 7:** Spectrum of pathologies in soft tissue tumor PVNS: pigmented villonodular synovitis; GCT: giant cell tumor.

Disease entity	Number (%)	Disease entity	Number (%)
Lipoma	4 (9.1)	PVNS	2 (4.5)
liposarcoma	2 (4.5)	Hemangioma	1 (2.3)
Myxoid liposarcoma	2 (4.5)	Glomus tumor	1 (2.3)
Benign spindle cell neoplasm	1 (2.3)	Angioleiomyoma	1 (2.3)
Pleomorphic sarcoma	1 (2.3)	Rhabdomyosarcoma	1 (2.3)
Nodular fasciitis	1 (2.3)	Neurofibromas	8 (18.1)
Myxofibrosarcoma	1 (2.3)	Schwannoma	2 (4.5)
Fibrosarcoma	3 (6.8)	Malignant nerve sheath tumor	1 (2.3)
Desmoplastic fibroblastoma	1 (2.3)	Morton neuroma	1 (2.3)
Fibromatosis	1 (2.3)	Undifferentiated sarcoma	4 (9.1)
Desmoid tumor	1 (2.3)	Squamous cell carcinoma	1 (2.3)
GCT of tendon	2 (4.5)		

**Figure 4:**
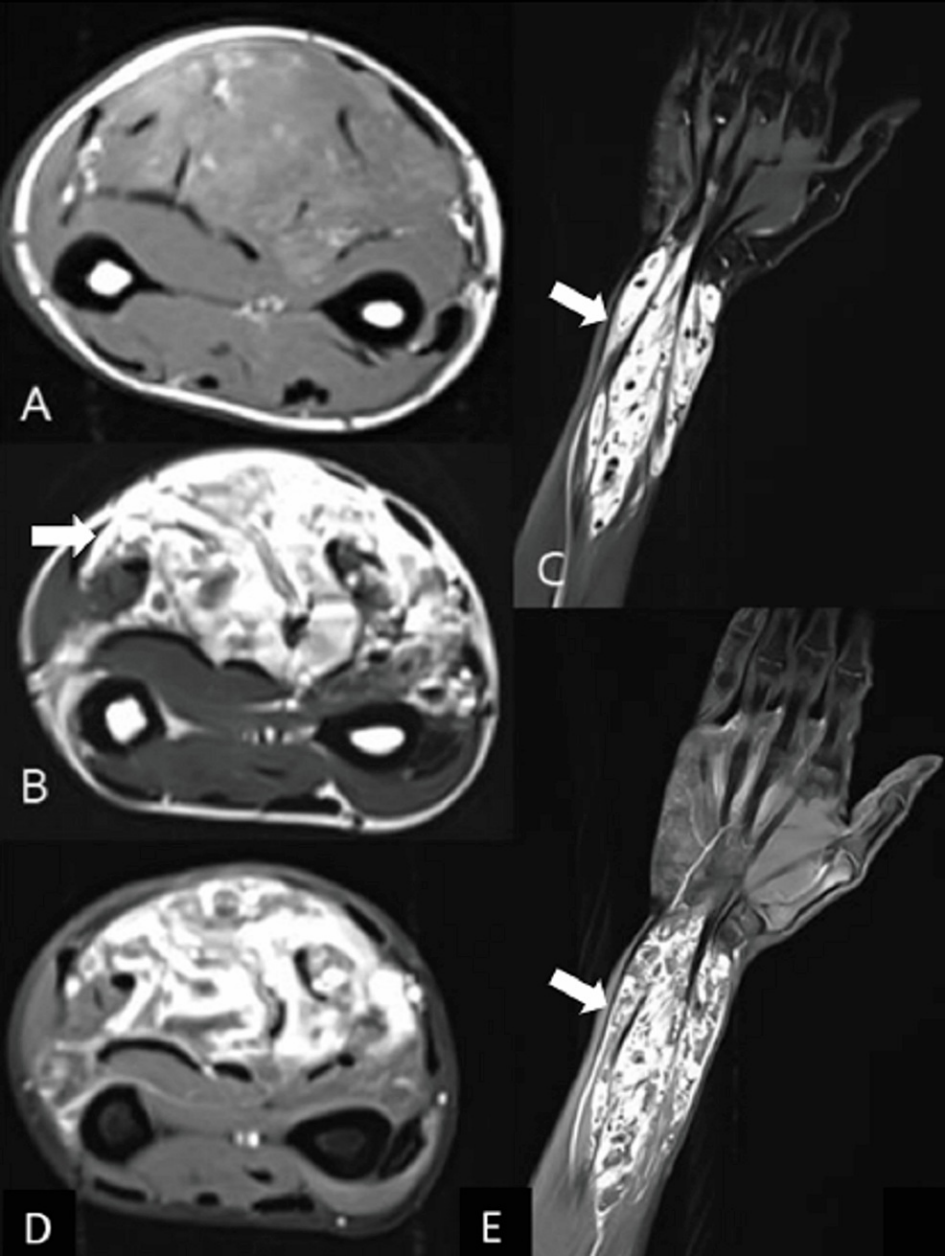
MRI of the forearm showing slow-flow venous malformation MRI of the right forearm in a 26-year-old female with progressively increasing swelling over the forearm for 10 years showing a large lobulated septated mass lesion seen in the subcutaneous, intermuscular, and intramuscular plane of the right forearm. The lesion appears heterogeneously iso-hyperintense to muscles on T1-weighted image (A), heterogeneously hyperintense on T2-weighted image (B) and short tau inversion recovery (STIR) (C, D) images, and shows heterogenous post-contrast enhancement with no enlarged arterial feeders or early draining veins (E). Findings are suggestive of slow-flow venous malformation.

Soft tissue tumor-like lesions were seen in 27 (38%) out of 71 patients. The most common tumor mimicked in our study was slow-flow vascular malformation, which was seen in nine (33%) out of 27 patients (Figure 4). The second most common pathology was actinomycosis, seen in four (14.8%) patients (Table 8).

**Table 8:** Spectrum of pathologies in soft tissue tumor-like lesions

Disease entity	Number	Percentage (n = 27)
Abscess	1	3.7
Actinomycosis	4	14.8
Baker’s cyst	2	7.4
Epidermal inclusion cyst	1	3.7
Gout	1	3.7
Hematoma	2	7.4
Hydatid cyst	1	3.7
Morel-Lavallée lesion	2	7.4
Parameniscal cyst	1	3.7
Prepatellar bursitis	1	3.7
Slow-flow vascular malformation	9	33.3
Ganglion cyst	2	7.4

Out of 44 patients with soft tissue tumors, 27 (61.4%) were proven to be benign, and 17 (38.6%) were found to be malignant on histopathological examination (HPE). Out of 27 patients with benign tumors, 14 (51.8%) were located in the deep plane while 13 (48.1%) were located in the superficial plane. Out of 17 patients with malignant tumors, 13 (76.4%) were located in the deep plane while four (23.5%) were located in the superficial plane (Table 9).

**Table 9:** Trans-spatial plane location of soft tissue tumors

Location	Benign (n = 27)	Malignant (n = 17)	Total (n = 44)
Superficial	13 (48.1)	4 (23.5)	17 (38.6%)
Deep (muscular, neurovascular, intermuscular plane)	14 (51.8)	13 (76.4)	27 (61.4%)
Total	27	17	44

Out of 44 soft tissue lesions in our study, 21 (47.7%) patients had tumor sizes of less than 5 cm, and the rest 23 (52.3%) had tumor sizes of more than 5 cm (47.7%) (Table 10). Out of 27 patients with benign tumors, 16 (59.2%) patients had <5 cm tumor size while 11 (40.7%) patients had >5cm tumor size. Out of 17 patients with malignant tumors, more (70.5%) patients had >5 cm tumor size than the five (29.4%) patients who had <5 cm tumor size. Out of 44 patients with soft tissue tumors, 31 (70.5%) patients had heterogenous enhancement patterns while homogeneous enhancement was seen in 11 patients (25%). Out of 27 benign tumor patients, 17 (62.9%) had heterogeneous enhancement, eight (29.6%) had homogeneous enhancement, and two (7.4%) showed no contrast enhancement. Out of 17 malignant tumor patients, 14 (82.3%) had heterogeneous enhancement while three (17.6%) showed homogeneous contrast enhancement. Out of 44 patients, 28 demonstrated diffusion restriction for which 15 (53.5%) were malignant and 13 (46.4%) were benign (Table 10).

**Table 10:** MRI characteristics of soft tissue tumors DWI: diffusion-weighted images.

Size	Benign (n = 27)	Malignant (n = 17)	Total (n = 44)
Less than 5 cm	16 (59.2)	5 (29.4)	21 (47.7%)
More than 5 cm	11 (40.7)	12 (70.5)	23 (52.3%)
Margins			
Smooth	19 (70.3)	5 (29.4)	24 (54.5%)
Irregular	0	3 (17.6)	3 (6.8%)
Lobulated	8 (29.6)	9 (52.9)	17 (38.6%)
MRI appearance			
No enhancement	2 (7.4)	0	2 (4.5%)
Homogenous	8 (29.6)	3 (17.6)	11 (25%)
Heterogeneous	17 (62.9)	14 (82.3)	31 (70.5%)
DWI			
Diffusion restriction absent	14 (51.8)	2 (11.7)	16 (36.3%)
Diffusion restriction present	13 (48.2)	15 (88.2)	24 (54.5%)

The table below (Table 11) reveals the correlation of the MRI diagnosis of soft tissue tumors with HPE considered as the gold standard. Out of 27 benign lesions proven on HPE, 25 were diagnosed as benign lesions and two were diagnosed as malignant lesions on MRI. Out of 17 HPE-proven malignant lesions, the diagnosis of malignant lesions was made on MRI in 15 patients while two lesions diagnosed as benign were proven malignant on HPE. In our study, the odds of a benign histopathological diagnosis for a tumor suspected to be benign by MRI were 93.75 times higher than the odds of a benign histopathological diagnosis for a tumor suspected to be malignant by MRI.

**Table 11:** MRI findings and histopathological correlation

	Final histopathological benign/malignant diagnosis		
MRI benign/malignant	Benign	Malignant	Total
Benign	25	2	27
Row %	92.59%	7.41%	100.00%
Col %	92.59%	11.76%	61.36%
Malignant	2	15	17
Row %	11.76%	88.24%	100.00%
Col %	7.41%	88.24%	38.64%
Total	27	17	44
Row %	61.36%	38.64%	100.00%
Col %	100.00%	100.00%	100.00%

## Discussion

In our study, male patients were more (69%) than females (31%) with a male-to-female ratio of 2.2:1, and a similar trend was seen in soft tissue tumors as well as in tumor-like lesions. This finding is similar to that reported by Tacikowska [11] and Chung et al. [12] who also reported a greater number of male patients than females in their studies of soft tissue tumors. The age of patients in our study ranged from six to 90 years. The most common group was young adults, with ages ranging from 20 to 40 years with a median age of 35 years and a mean age of 39.7 ± 19.2 years. The findings are similar to those reported by Tacikowska who also reported 110 patients in the age group of 16-84 years [11].

In our study of 71 patients, the most common complaint was swelling seen in 57 patients (76%). Forty-two out of our 71 patients had complained of pain associated with swelling with 15 patients having asymptomatic swelling. Patients also presented with other symptoms like localized redness of the region (10%), fever (6%), and a tingling sensation in the region (8%). The clinical history can help in the evaluation of the soft tissue lesions based on information such as location, duration of the swelling, presence or absence of associated pain, rate of growth, and the presence of multiple lesions [12,13].

Out of 71 cases, we found 44 cases of soft tissue tumors whereas the remaining 27 cases mimicked soft tissue tumors. PNSTs were the most common soft tissue masses and were seen in 10 out of 44 patients (22.7%). Chung et al. conducted a study of 266 patients with histologically proven soft-tissue tumors of the extremities; they found lipoma as the most common subgroup seen in 39 (24%) patients followed by schwannoma seen in 27 (16%) patients [14].

Out of 44 cases, soft tissue tumors in 31 patients were found in the lower extremity where the most common location was the thigh (12 patients) followed by the leg (seven patients). Out of 12 patients with soft tissue tumors located in the thigh, nine were found to be malignant. In the remaining 13 patients, soft tissue tumor was located in the upper extremity with the most common location being the arm seen in six cases. Out of these six cases, four were benign tumors and two were malignant tumors. In their study of lipoma and sarcoma in 428 patients, Rydholm et al. found that lesions located in the thigh irrespective of depth and size were more likely to be sarcoma [3]. The majority of the lesions that were located in the superficial plane (17 of 44 or 38.6%) were benign (13 of 17), with the most common being benign neurogenic tumor. Out of 17 malignant soft tissue tumors, 13 were located in the deep plane and only four were located in the superficial plane. Church et al. stated that smaller and superficially located lesions (less than 5 cm) are usually benign whereas the lesions that are larger (more than 5 cm) and located in deeper soft tissue planes are more likely to be malignant [15]. However, this may not always hold true, and almost one-third of soft tissue sarcomas can occur in a superficial location [16].

We found that out of 44 cases of soft tissue tumors, 21 cases were smaller in size (less than 5 cm), and 23 cases were larger in size (more than 5 cm). Out of 21 cases that were less than 5 cm in size, 17 were benign. Twelve out of 17 malignant tumors were more than 5 cm in dimension. Previous studies have documented a higher risk of malignancy in lesions having a size greater than 5 cm [15,17,18]. Tung et al. reported that the sensitivity and specificity of tumor malignancy assessment for lesions exceeding 5 cm were 74% and 59%, respectively [18]. Margin characterization of lesions on MRI is another criterion that can be used for classifying the lesion as benign or malignant lesions. We found that the tumors with smooth margins were mostly benign, seen in 19 out of 27 patients. All three lesions having irregular margins in our study were found to be malignant. Tumors with lobulated margins had an inclination toward malignancy seen in nine of 17 patients, whereas in benign lesions, lobulated margins were seen in eight of 27 patients. Vadapalli et al. stated that tumors with irregular margins were likely to be malignant while the tumors having smooth margins were usually benign [19]. However, specific data for the characterization of the lesion into benign and malignant based on the margin of the lesion is not well studied in the literature.

According to the contrast enhancement pattern in our study, most of the tumors (31 of 44 or 70%) showed heterogeneous enhancement, 11 (25%) cases showed homogeneous enhancement, and two (4%) cases showed no enhancement. The tumors with no or homogeneous contrast enhancement were found to be benign seen in 10 patients. Malignant tumors showed predominantly heterogeneous enhancement seen in 14 of 17 cases. In their study of 95 patients with soft tissue masses, Berquist et al. stated that the presence of homogeneous signal enhancement was unusual in malignant lesions with at least a few heterogeneous signal enhancements usually present in the malignant lesions [20]. However, they also reported the presence of heterogeneity in benign lesions, which suggests that enhancement pattern analyses are more useful for the diagnosis of malignant lesions than for benign lesions. Vadapalli et al. stated that sarcomas showing heterogeneous signal intensity and contrast enhancement were likely to be malignant with the tumors showing homogeneous signal and enhancement were usually benign [19]. A scoring system based on the characteristics of conventional US and MRI was established by the regression model in a study by Shu et al. of 120 pathologically confirmed instances of soft tissue tumors (71 cases of malignant lesions and 49 cases of benign lesions) [21]. They concluded that the features of the margin, diameter, and peripheral tissue enhancement offer helpful information for differentiating between benign and malignant soft tissue masses.

The composition of the lesion helps in the imaging characterization of lesions on MRI as the different matrix components show different signal intensity patterns in various MRI sequences, which helps to establish the pathological diagnosis. Adipocyte-containing masses appear hyperintense on T1-weighted images (T1WI) and T2-weighted images (T2WI) with suppression noted on the fat-saturated sequence. A lesion with fluid appears hypointense on T1WI and hyperintense on T2WI with no enhancement noted on post-contrast sequences. The masses with hemorrhage have different signal characteristics depending on the stage of hemorrhage with areas of blooming noted on gradient sequences. A lesion with a predominantly fibrous component appears hypointense on both T1WI and T2WI with delayed enhancement. Wu et al. stated that tissue matrix plays a role in the imaging appearance of soft tissue lesions on different MRI sequences with fat, methemoglobin, proteinaceous fluid, and melanin causing T1 shortening and appearing hyperintense on T1WI [1]. Lesions containing dense calcifications, areas of fibrosis, and hemosiderin appear hypointense on T2WI while fluid-filled lesions and solid lesions appear hyperintense on T2WI. Similar tissue matrices were also seen in various soft tissue lesions in our study, depending on the tissue composition (Tables 3, 4).

In our study, the appearance of lesions on other MRI sequences like DWI and GRE sequences was also assessed. Also, in our study, diffusion restriction was present in 15 of 17 or 88.2% of malignant soft tissue tumors and 13 of 27 or 48.1% of benign soft tissue tumors. In their study of DWI in soft tissue tumors, Pekcevik et al. found that though some overlap existed between benign and malignant tumors on DWI, the addition of DWI to routine MRI improved the diagnostic accuracy [22].

In a study conducted by Chung et al. on 266 patients with histologically proven soft tissue tumors of the extremities, the lesions were analyzed according to depth (superficial or deep), size (<50 and ≥50 mm), and signal intensity (homogeneous or heterogeneous) on T2WI, to determine the ability of each to predict benign and malignant tumors [14]. They found that combined systematic analysis based on signal intensity, size, and depth provides higher diagnostic value in malignancy with 64% sensitivity, 85% specificity, and 77% accuracy. Chung et al. recommended a simplified systematic imaging approach based on signal intensity, size, and depth, to distinguish between benign and malignant soft-tissue tumors [14].

The spectrum of lesions included in the soft tissue tumor-like lesion was abscess, actinomycosis, gout, hematoma, Morel-Lavallée lesion, prepatellar bursitis, slow-flow vascular malformation, cystic lesions like Baker’s cyst, epidermal inclusion cyst, parameniscal cyst, and hydatid cyst. Among the soft tissue mimics, slow-flow vascular malformations were the most common, seen in nine of 27 patients while actinomycosis was the second most commonly seen in four of 27 patients. All the slow-flow vascular malformation cases had hyperintense signals on T2WI and STIR images. Few lesions showed fluid-fluid levels due to slow venous flow. The majority of these lesions (five of nine) were located in the superficial plane with multicompartmental extent noted in the rest of the lesions. All of these lesions had heterogeneous post-contrast enhancement with contrast filling in the delayed scan. Phleboliths were also noted in the lesions, which further helped in diagnoses of slow-flow vascular malformation. Laor stated similar characteristic features on MRI with the hyperintense appearance of the lesion on T2WI with prompt contrast filling. Loss of signal due to phlebolith was seen in the lesion [23]. Vadapalli et al. and Laor also stated that based on clinical presentation, location, and characteristic imaging findings, the soft tissue tumor can be differentiated from common soft tissue tumor-like lesions such as arterio-veno-lymphatic malformations, hematoma, actinomycosis, and various cystic lesions like ganglion cyst and synovial cysts [19,23]. Out of the four cases of actinomycosis in our study, three were located in the superficial plane and showed a diagnostic dot-in-circle radiological sign. This dot-in-circle sign was characterized on T2WI by a small round hyperintense area composed of granulation tissue surrounded by a hypointense rim, which represents fibrous septa with a central hypointense dot due to susceptibility loss by fungi and is a highly specific sign for this lesion [24].

In our study, the analysis of the benign and malignant soft tissue tumors was done based on imaging characteristics on MRI, and HPE correlation was obtained. We found that the odds of a benign histopathological diagnosis for a tumor suspected to be benign by MRI were 93.75 times higher than the odds of a benign histopathological diagnosis for a tumor suspected to be malignant by MRI. A similar study by Berquist et al. reported that the accuracy for diagnosis of benign and malignant soft tissue tumors was 85-90% and found MRI has a 94% negative predictive value, i.e., a lesion is not malignant [20]. In a comparative study between MRI and HPE of a group of 69 surgical patients, Tacikowska concluded that agreement of results between MRI and histopathology was seen in 29 patients and disagreement was seen in five patients, and inferred MRI is useful in the diagnosis of soft tissue sarcoma [11].

**Figure 5:**
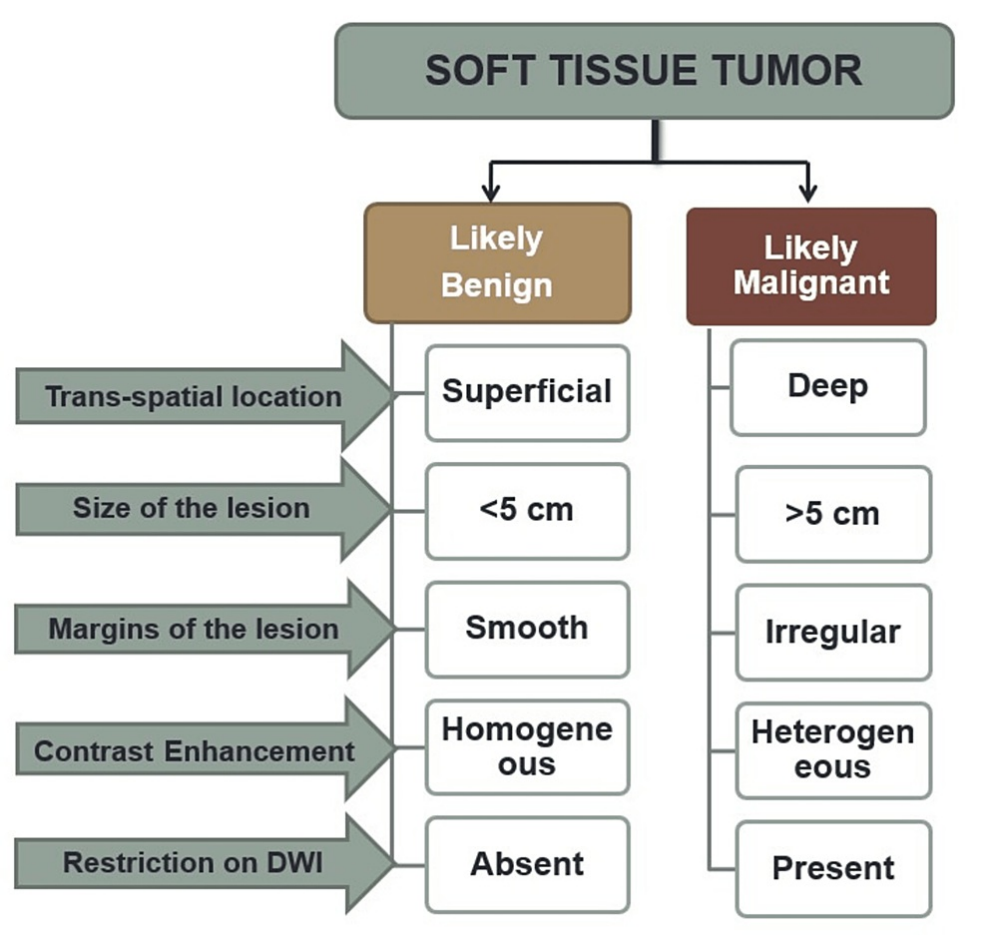
Systematic MRI analysis approach in the benign and malignant soft tissue tumors DWI: diffusion-weighted imaging.

We found that a systematic imaging analysis approach helps in the characterization of the lesion and hence helps to differentiate a benign lesion from a malignant lesion (Figure 5). The major parameters that help are the depth of the lesion, its location, size, margins, and the contrast enhancement patterns analyzed in our study. Analysis of signal intensity to determine tissue matrix, use of DWI imaging, plus some specific signs like a dot in a circle, split fat, and target signs further add diagnostic values in the assessment of soft tissue tumors and tumor-like lesions.

Limitations

A limitation of MRI is that it requires extended time for the investigation to be completed, which is difficult in the case of children as well as in the elderly who require a dose of sedation for the scan to be conducted. It is also not possible in the case of claustrophobic patients. Another obstacle in imaging is the sparse availability of MRI scanners in remote areas. Our study had a further limitation due to our small sample size of soft tissue tumors, which might not be representative of the soft tissue tumor distribution.

## Conclusions

MRI is useful in the evaluation of various soft tissue masses. MRI offers information on the characteristic of the mass, its extent and relationship to the adjacent structures, multiplicity, composition, and enhancement pattern that depicts its vascular details. A systematic imaging analysis approach helps in the characterization of the lesion and hence helps to differentiate a benign lesion from a malignant lesion. The major parameters that help are the depth of the lesion, its location, size, margins, and contrast enhancement patterns. MRI helps in differentiating various mimics of soft tissue tumors, which might be mistaken for soft tissue tumors based on clinical presentation, location, and various specific imaging characteristics seen on MRI.
